# Temperature and oxygen adsorption coupling effects upon the surface tension of liquid metals

**DOI:** 10.1038/s41598-019-43500-3

**Published:** 2019-05-08

**Authors:** Aïmen E. Gheribi, Patrice Chartrand

**Affiliations:** CRCT - Polytechnique Montréal, Chem. Eng., Box 6079, Station Downtown, Montréal, Qc H3C 3A7 Canada

**Keywords:** Surfaces, interfaces and thin films, Chemical physics, Surfaces, interfaces and thin films, Chemical physics

## Abstract

An accurate knowledge of the surface tension of liquid metals is critical for many theoretical and practical applications, especially in the current context of emerging growth of nanotechnology. The surface tension and its temperature dependence are drastically influenced by the level of impurities in the metal such as oxygen, sulphur or carbon. For this reason, experimental surface tension data of metals reported in literature are scattered. Strictly speaking, when referring to the surface tension of liquid metals, both variables temperature and oxygen content must be specified. There exists no clear formalism describing the coupling effect temperature and the oxygen content upon the surface tension of liquid metals. The aim of this work is to fill this gap. A thermodynamically self-consistent formulation for the surface tension of liquid metals and semiconductors as a function of temperature and oxygen content is established. According to the proposed formalism, a reliable expression for the surface tension of pure and oxygen saturated metals is then derived. The proposed model is found to be in good agreement with available experimental data, showing a good predictive capability. Aluminium is chosen and thoroughly evaluated as a case study, due to its very high sensitivity to oxygen level. Its surface tension is explicitly formulated as a function of temperature and oxygen content.

## Introduction

Although the surface tension of liquid metals has been extensively studied for nearly a century, both from an experimental and a theoretical point of view, there is still no clear value of the surface of tension liquid metals. In general, the surface tension decreases linearly with temperature:

1 $$\sigma (T)={\sigma }^{0}({T}_{m})-\sigma ^{\prime} (T-{T}_{m})$$

where *σ*^0^ and $\sigma ^{\prime} ={(\partial \sigma /\partial T)}_{{T}_{m}}$ denote respectively the surface tension and its temperature derivative, assumed to be constant, at *T*_*m*_ (melting temperature). Equation 1 has proven reliable in a wide range of temperature, a least up to *T*_*C*_/2, *T*_*C*_ being the crtical temperature. The experimental values of both *σ*^0^ and *σ*′ reported in the litterature are often scattered, not only because of the errors inherent to experimental methods, but also because they are strongly influenced by the presence of impurities at the surface of the liquid. For instance, the reported value of *σ*^0^ for aluminium is very often: 0.85 N.m^−1 ^^1^. However, this value is not for pure aluminum but for aluminium saturated in oxygen. Indeed, because Al and O have a strong chemical affinity, a few ppm of O_2_ reacts with Al to form an Al_2_O_3_ monolayer at the surface. The oxide monolayer causes a drastical decrease of the surface tension, from 1.05 ± 0.05 N.m^−1^ to 0.85 ± 0.05) N.m^−1 ^^2,3^. This example reflects the surface tension’s extreme sensitivity to oxygen, and more generally to impurities such as S, C, P. This could impact the alloy and process design of many industrial applications, in particular those in a controlled atmosphere. In the literature, several studies report a value of *σ*′, however no consensus can be established as there is a dispersion of over 100% in reported data^1^. Inconsistencies in experimental data cannot be harmonized by a critical assessment as the impurity content must be considered as a variable. The primary purpose of this work is to establish a clear and reliable formalism to describe the surface tension as a function of both temperature and oxygen content.

In our recent work, considering the Gibbs adsorption isotherm concept, we have shown that the oxygen content (*x*_*O*_) dependence upon surface tension can be by first degree approximation assumed to be directly proportional to the value of the surface tension at a given temperature: (∂*σ*/∂*x*_*O*_) ∝ *σ*. By integration, the following expression has be formulated^4^:

2 $$\sigma (T,{x}_{O})={\sigma }_{pure}(T)[1-{\lambda }_{O}^{sat}{{\rm{\Gamma }}}_{O}^{sat}(1-{e}^{-{\xi }_{O}\frac{{x}_{O}}{{x}_{O}^{sat\mathrm{.}}(T)}})]$$

where ${{\rm{\Gamma }}}_{O}^{Sat\mathrm{.}}$ and ${x}_{O}^{sat\mathrm{.}}$ are respectively the full coverage oxygen adsorption at the liquid surface and the oxygen content at full coverage (saturation). Both parameters ${\lambda }_{O}^{sat}$ and *ξ*_*O*_ are universal constants, i.e. identical for all elements. Their values were determined by Gheribi *et al*.^4^ as: ${\lambda }_{O}^{sat}=16078$ and *ξ*_*O*_ = 7.422. ${{\rm{\Gamma }}}_{O}^{sat\mathrm{.}}$ can be determined from the lattice constants of the corresponding metal-oxyde monoxide using the well established Kozakevitch’s approximation given by^5^:

3 $${{\rm{\Gamma }}}_{o}^{sat\mathrm{.}}=\frac{{n}_{O}}{A\cdot {N}_{A}}$$

where *A* is the mesh surface, *n*_*O*_ the number of oxygen at the mesh surface and *N*_*A*_ the Avogadro number. For monoxide, *n*_*O*_ = 2. In practice, *A* is defined as ${({n}_{c}\cdot {V}_{m},o\cdot {N}_{A}^{-1})}^{\frac{2}{3}}$, with *V*_*m*,*o*_ and *n*_*c*_ are respectively the molar volume of the monoxide at standard temperature (298.15 K) and the number of atoms per unit cell^6^. In a nutshell, in this formalism (Eq. 2) there is no adjustable parameter, provided that *σ*^0^(*T*) and ${x}_{O}^{sat\mathrm{.}}$ are known. ${x}_{O}^{sat\mathrm{.}}$ is determined from phase equilibria and crystallographic data and in general it is known with an appreciable accuarcy while *σ*^0^(*T*) is not well defined for most elements, especially those with a significant reactivity with oxygen. Indeed, most of the time, the reported values for *σ*^0^(*T*) are underestimated, as they are related to experiments performed with contaminated samples (with non-metallic impurities, in particular oxygen). In practice, there is no clear formalism describing the surface tension of pure elements as a function of temperature but also the temperature-impurities content coupling effect upon the surface tension of metals. Naturally, several assessements and recommended parameters for Eq. 1 can be found in the litterature, see e.g.^1,7–10^, however none of them clearly recommends values for temperature dependent surface tension for pure metals.

## Thermodynamically consistent formulation of the temperature dependence of the surface tension

Let us consider a pure element, from a thermodynamic point of view. The temperature derivative of the surface tension defines the excess surface entropy, Δ*S*^*s*^^11^:

4 $${(\frac{\partial {\rm{\Delta }}{S}^{s}}{\partial A})}_{T,P}=-\,{(\frac{\partial \sigma }{\partial T})}_{A,P}=-\,{(\frac{\partial }{\partial T}{(\frac{\partial {\rm{\Delta }}{F}^{s}}{\partial A})}_{T})}_{A}$$

where A and Δ*F*^*s*^ are respectively the surface and the excess surface Helmholtz free energy. The molar surface, *A*_*m*_, is expressed as^12^ ${A}_{m}=L\cdot {N}_{A}^{\mathrm{1/3}}\cdot {V}_{m}^{\mathrm{2/3}}$, where L is a factor taking into account the packing of the liquid, *N*_*A*_ is the Avogadro number and *V*_*m*_ the molar volume. L lies in general between 1.04 and 1.12, however the generic value of 1.09 is generally used, corresponding to the packing factor of close-packed structures. Then:

5 $${(\frac{\partial \sigma }{\partial T})}_{A,P}=-\,\frac{1}{L{N}_{A}^{\mathrm{1/3}}}{(\frac{\partial }{\partial T}{(\frac{\partial {\rm{\Delta }}{F}_{m}^{s}}{\partial {V}_{m}^{\mathrm{2/3}}})}_{T})}_{A}$$

Without loss of generality it can be assumed that the surface and the bulk Helmholtz free energy are proportional, i.e. ${F}_{m}^{s}\propto {\rm{\Delta }}{F}_{m}$. Neglecting the difference between the bulk and the surface electronic structure, this proportionality can be expressed as^13^:

6 $${\rm{\Delta }}{F}_{m}^{s}=(\frac{{Z}_{S}-{Z}_{B}}{{Z}_{B}}){F}_{m}$$

where *Z*_*B*_ and *Z*_*S*_ are respectively the bulk and the surface coordination. Then we denote the ratio (*Z*_*B*_ − *Z*_*S*_)/*Z*_*B*_ by *β*. Thereafter, given that the Maxwell relations define (∂^2^*F*_*m*_/∂*V*∂*T*) as the product: *α*_*V*_*B*_*T*_^14^ where *α*_*V*_ and *B*_*T*_ are the volumetric thermal expansion and the isothermal bulk modulus, Eq. 5 can be rewritten as:

7 $${(\frac{\partial \sigma }{\partial T})}_{A,P}=-\,\frac{3\beta }{2L{N}_{A}^{\mathrm{1/3}}}{V}_{m}^{\mathrm{1/3}}{\alpha }_{V}{B}_{T}$$

Note that the product ${\alpha }_{V}\cdot {B}_{T}$ is defined as the thermal pressure coefficient. For liquid and solid metallic or semiconductors elements, the thermal pressure coefficient is nearly independent of temperature^15^, thus (∂*σ*/∂*T*)_*A*,*P*_ is a constant that can be written as ${\sigma }_{pure}^{^{\prime} }$. In general, for liquid metals, the generic average of *L* = 1.091 is used^16^, then one can define:

8 $${\sigma }_{pure}^{^{\prime} }(T)=-\,1.628\times {10}^{-8}\beta \,{V}_{m}^{\mathrm{1/3}}{\alpha }_{V}{B}_{T}$$

where *β* characterises the ratio of “broken bonds” at the surface compared to the bulk and should be specific for each element as, contrary to crystals, the bulk coordination of liquid metals varies between elements. For FCC and BCC crystals, it is well known that *β* = 0.25. For bulk liquid metals the average coordination number is 10.35 ± 0.06^17,18^. If, as suggested by Kaptay *et al*.^19^, we assume that surface liquid metals are structured similarly to the (1 1 1) FCC lattice plane with *Z*_*S*_ = 9, the average value of *β* should be as follows:

9 $$\langle \beta \rangle \simeq 0.132\pm 0.045$$

This value is close to *β* = 1/6 and *β* = 0.174 ± 0.023 proposed respectively by Oriani^20^ and Kaptay *et al*.^19^.

As mentioned before, the surface tension of pure metals at melting temperature, ${\sigma }_{pure}^{0}$, is not precisely known. In fact, little data is available. One of the advantages of the Gheribi *et al*. formalism^4^ is that the surface tension of pure liquid metals and its temperature dependence can be deduced directly from those of oxygen saturated ones. Indeed, according to Eq. 2:

10 $$\frac{{\sigma }_{pure}({T}_{m})}{{\sigma }_{sat\mathrm{.}}({T}_{m})}=\frac{{\sigma }_{pure}^{^{\prime} }({T}_{m})}{{\sigma }_{sat\mathrm{.}}^{^{\prime} }({T}_{m})}=\frac{1}{1-{\lambda }_{O}^{sat}{{\rm{\Gamma }}}_{O}^{sat}}$$

where *σ*_*sat*._ and ${\sigma }_{sat\mathrm{.}}^{^{\prime} }$ are the surface tension of the oxygen saturated liquid metal and its temperature derivative assumed constant. In our recent publication^4^, we demonstrated the reliability of Eq. 9 by predicting the surface tension drop between pure and oxygen saturated metals for several case studies. The fact that in the present formalism the product ${\lambda }_{O}^{sat}{{\rm{\Gamma }}}_{O}^{sat}$ governs the variation of both *σ* and *σ*′ at oxygen saturation is not surprising. Indeed, the model describing the oxygen content effect upon the surface tension (Eq. 2) originates from the assumption that (∂*σ*/∂*x*_*O*_) ∝ *σ*. From a thermodynamic point of view, the excess surface entropy has two contributions: (i) a vibrational contribution due to the difference of quasi-lattice vibration at the bulk and surface, ${\rm{\Delta }}{S}_{vib}^{s}$, and (ii) a configurational contribution, ${\rm{\Delta }}{S}_{conf\,.}^{s}$, due to the presence of impurities at the surface. Then, one can define:

11 $${(\frac{\partial \sigma }{\partial T})}_{A,P}=-\,\frac{({\rm{\Delta }}{S}_{vib\mathrm{.}}^{s}+{\rm{\Delta }}{S}_{conf\mathrm{.}}^{s})}{{A}_{m}}$$

Thereby, the two contributions of excess surface entropy can be defined by identifying Eq. 11 to Eq. 7 and Eq. 10:

12 $$\begin{array}{rcl}{\rm{\Delta }}{S}_{vib}^{s} & = & \frac{3}{2}\beta {V}_{m}{\alpha }_{V}{B}_{T}\\ {\rm{\Delta }}{S}_{conf\,.}^{s} & = & -{\lambda }_{O}^{sat}{{\rm{\Gamma }}}_{O}^{sat}{\rm{\Delta }}{S}_{vib}^{s}\end{array}$$

The excess vibrational surface entropy is positive while the excess configurational surface entropy is negative. This is in agreement with the principles of statistical physics^12^. In the present formalism, both the vibrational and configurational excess entropies are correlated. This is also consistent with the Skapski^12,13^ formalism of the surface entropy. Indeed, from statistical mechanics principles, Skapski derived an expression for the excess configurational and vibrational surface entropy, demonstrating that both contributions can be approximated by functions depending only on the ratio between the average bulk and surface coordination numbers. This can be explained by the fact that the main contribution for the difference between the thermodynamic properties of bulk and surface is the difference between the coordination. Eq. 12 is, to some extent, consistent with the Skapski formalism as the vibrational and configurational excess entropies are intercorrelated via *β*.

In a nutshell, according to the present formalism, one can **predict**:

- the temperature dependence of the surface tension of pure and oxygen saturated liquid metals
- the surface tension of pure liquid metals at melting point from its oxygen saturated surface tension
- the surface tension of liquid metals as a function of temperature and oxygen content from the knowledge of the surface tension of oxygen saturated metals at the melting point

It should be noted that the measure of the solubility of oxygen in liquid metals can be either obtain from e.m.f. measurements^21^ or by electrochemical methods such as described in^22,23^.

## Results and Discussion

Let us now validate the formalism. For 20 liquid metals, we compare the experimental temperature dependence of the surface tension of pure or “nearly” pure elements with predictions by Eq. 8. The purest experimental data were chosen as samples for experiments. In addition, chosen experimental data must have been reported in a quite large range of temperatures, ≳200 K. To calculate the thermal pressure coefficient, we have considered the critically assessed density, thermal expansion, heat capacity (C_*P*_) and velocity of sound (C_0_) (references are given in Table 1). The density of liquid metals varies linearly with temperature: *ρ*(*T*) = *ρ*^0^ − *ρ*′(*T* − *T*_*m*_). Then, at the melting temperature, thermal expansion is: *ρ*′/*rho*^0^ and the isothermal bulk modulus is deduced from the velocity of sound according to the relation: ${B}_{T}=({c}_{0}^{2}\rho )/(1+{\alpha }_{V}\gamma T)$, where *γ* is the Grüneisen parameter defined as $\gamma =({\alpha }_{V}{V}_{m}^{2}{C}_{p})/({C}_{0}^{2}M)$, *M* being the molecular weight.

**Table 1:** Critically assessed density (*ρ*), density temperature dependence coefficient *ρ*′, average velocity of sound (*C*_0_), heat capacity at constant pressure, (*C*_*P*_).

#	*T*_*m*_	*ρ* ^0^	*ρ*′	*C*_0_	*C*_*P*_	10^4^ × *α*_*V*_/K	*B*_*T*_	*σ*(*Exp*.)	10^4^*σ*′(*Exp*.)	10^4^*σ*′(*Pred*.)
		kg/m^3^	kg/(m^3^.K)	m/s	J/(mol.K)		GPa	J/m^2^	J.(m^2^.K)	J.(m^2^.K)
Si	1683	2550	0.26^*a*^	3920^*f*^	29.20^*g*^	1.04	30.95	0.83^*h*^	−1.00	−1.53
Ni	1727	7861	0.99^*b*^	4047^*f*^	43.08^*g*^	1.26	80.02	1.85^*i*^	−3.64	−4.22
Fe	1811	7035	0.93^*c*^	4200^*f*^	46.00^*g*^	1.32	74.35	1.93^*j*^	−4.00	−4.19
Sn	505	6979	0.65^*d*^	2464^*f*^	29.69^*g*^	0.93	38.30	0.61^*k*^	−1.70	−1.98
Cu	1356	7997	0.82^*d*^	3440^*f*^	32.84^*g*^	1.02	71.41	1.40^*l*^	−3.30	−3.14
Bi	544	10028	1.21^*b*^	1640^*f*^	30.49^*g*^	1.21	23.52	0.38^*m*^	−0.70	−1.68
Ag	1234	9264	0.88^*b*^	2790^*f*^	33.47^*g*^	0.95	56.48	0.96^*n*^	−1.85	−2.60
Co	1766	7827	0.94^*a*^	4031^*f*^	40.46^*g*^	1.20	79.43	1.89^*o*^	−3.30	−4.00
Al	934	2377	0.31^*c*^	4561^*f*^	31.75^*g*^	1.31	38.57	1.02^*p*^	−2.74	−2.44
Cd	593	8008	1.25^*a*^	2256^*f*^	29.71^*g*^	1.56	31.86	0.66^*q*^	−2.50	−2.58
Ga	303	6077	0.61^*a*^	2873^*f*^	28.47^*g*^	1.01	47.24	0.72^*r*^	−0.68	−2.30
Ge	1211	5600	0.55	2693^*f*^	27.61^*g*^	0.98	33.01	0.66^*s*^	−1.56	−1.64
In	430	7022	0.76^*a*^	2337^*f*^	29.48^*g*^	1.09	34.58	0.57^*t*^	−0.90	−2.05
K	337	838	0.23	1876^*f*^	32.16^*g*^	2.77	2.66	0.12^*u*^	−0.62	−0.57
La	1203	5940	0.61^*e*^	2030^*f*^	34.31^*g*^	1.03	20.23	0.75^*v*^	−1.00	−1.28
Na	371	927	0.23^*e*^	2526^*f*^	31.87^*g*^	2.48	5.35	0.21^*w*^	−0.50	−0.83
Pb	661	10656	1.24^*b*^	1821^*f*^	30.45^*g*^	1.16	29.90	0.48^*x*^	−2.40	−2.01
Ti	1958	4140	0.15^*e*^	4309^*f*^	47.24^*g*^	0.36	64.31	1.56^*y*^	−0.62	−1.12
Au	1336	17310	1.34^*e*^	2568^*f*^	30.96^*g*^	0.77	85.37	1.19^*n*^	−2.51	−3.20
Sb	904	6467	0.61^*b*^	1900^*f*^	31.38^*g*^	0.94	20.99	0.38^*z*^	−0.84	−1.13

Both thermal expansion (*α*_*V*_) and isothermal bulk modulus (*B*_*T*_) are deduced from *ρ*, *ρ*′, *C*_0_ and *C*_*P*_ (see text). The predicted (*Pred*.) temperature dependence coefficient of pure liquid metals (*σ*′) is given in comparaison with the experimental ones (*Exp*.) and surface tension at melting point (*σ*) for supposedly pure metals. References are as follows: ^*a*^^56^, ^*b*^^57^, ^*c*^^58^, ^*d*^^59^, ^*e*^^60^, ^*f*^^24^, ^*g*^^25^, ^*h*^^61^, ^*i*^^62^, ^*j*^^63^, ^*k*^^2^, ^*l*^^64^, ^*m*^^65^, ^*n*^^66^, ^*o*^^67^, ^*p*^^49^, ^*q*^^68^, ^*r*^^69^, ^*s*^^70^, ^*t*^^45^, ^*u*^^71^, ^*v*^^72^, ^*w*^^73^, ^*x*^^74^, ^*y*^^75^, ^*z*^^76^. Note that references for *σ* and *σ*′ are identical as they are from the same set of experimental data.

**Figure 1:**
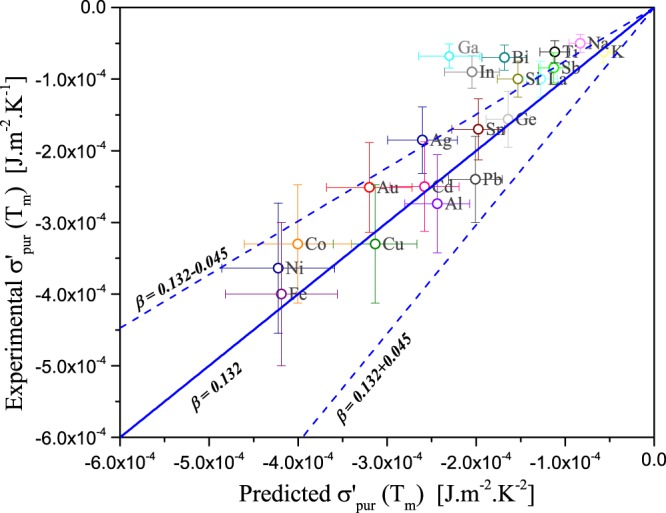
Parity plot representing predicted versus experimental temperature dependence coefficients of the surface tension (${\sigma }_{pure}^{^{\prime} }$) for 20 pure liquid metals. The solid line represents the predicted ${\sigma }_{pure}^{^{\prime} }$ via Eq. 8 with the average value of *β* = 0.132 while the two dash lines represent the predicted ${\sigma }_{pure}^{^{\prime} }$ with the upper and lower limits of *β* as defined by Eq. 9. The error bars are determined to be ±25% for experimental ${\sigma }_{pure}^{^{\prime} }$ and ±15% for predicted values.

Table 1 presents the predicted and the experimental temperature dependence coefficients of the surface tension for 20 pure liquid metals, along with the corresponding surface tension at melting temperature. The physical properties needed to calculate the thermal pressure coefficient (*α*_*V*_*B*_*T*_) and thus to predict ${\sigma }_{pure}^{^{\prime} }$ are also reported. Figure 1 compares the predicted and experimental ${\sigma }_{pure}^{^{\prime} }$ for 20 liquid metals for which reliable experimental data were available. Conservative error bars were assumed to be 25% and 15% respectively for experimental and predicted ${\sigma }_{pure}^{^{\prime} }$. Error bars for predicted ${\sigma }_{pure}^{^{\prime} }$ come from the uncertainty in *ρ*′ but especially in *C*_0_ as the uncertainty in *C*_0_ measurements is important^24^ (10–15%). In general, the agreement between experimental and predicted ${\sigma }_{pure}^{^{\prime} }$ is satisfactory, most of the data are within the model limits represented by the upper and lower values of *β*. This indicates the reliability of the proposed formalism, i.e. a linear relationship between the temperature dependence coefficient of the surface tension and the thermal pressure coefficient (Eq. 8). The deviation from between the predictions made with *β* = 0.132 and experimental data could be explained by a fluctuation of surface coordination numbers. Indeed, in all likelihood, *beta* should be specific to each element as *Z*_*S*_ and *Z*_*B*_ varies from an element to an other. The model accuracy could be improved if surface coordination numbers data were available. For now, the average value of *β* = 0.132 is at first glance satisfactory for all liquid metals. It is interesting to note that for Bi, Ga and In the value of *β* seems to be smaller than that of liquid metals indicating that for these elements, *β* is smaller, close to 0.066. In other words, for these three elements, the difference between *Z*_*S*_ and *Z*_*B*_ is less pronounced than other liquids metals, a difference of about 6.6%. A better estimation of *β* for each element is an important issue to explore in the near future as it could improve the model accuracy. That would however require an accurate prediction of surface coordination numbers for each element, via atomistic simulations.

In summary, according to the proposed formalism, **only** the surface tension value at oxygen saturation and the maximum oxygen solubility as a function of temperature are required to predict the surface tension as a function of both temperature and oxygen content. The calculation procedure can be written as follows:

1. Calculate ${{\rm{\Gamma }}}_{O}^{sat}$ via Eq. 3 and select from literature (or from thermodynamic database) critically assessed values of ${x}_{O}^{sat\mathrm{.}}$ as a function of temperature
2. Select from literature critically assessed ${\sigma }_{O}^{sat\mathrm{.}}$ at meting or reference temperature
3. Predict *σ*_*pure*_ via Eq. 10
4. Predict ${\sigma ^{\prime} }_{pure}$ via Eq. 8
5. Deduce $${\sigma ^{\prime} }_{sat\mathrm{.}}$$
6. Parametrize Eq. 2 and represent *σ*(*T*, *x*_*O*_).

As a case study for this procedure, we propose to examine the surface tension of aluminium. Aluminium is the second most produced metal in the world. Controlling the surface tension of aluminium and aluminium alloys is of primary importance in many industrial application. Even though it is well known that oxygen decreases drastically the surface tension of aluminium, there exists in the literature no clear formulation of the surface tension of aluminium as a function of both temperature and oxygen content. Very little of oxygen, (~5 ppm in general), is enough to saturate the surface of the liquid with an oxide monolayer. As a result, most experimental data reported in the literature are those of oxygen saturated aluminium. In practice, it is very likely that the oxygen content in the atmosphere is enough to saturate the aluminium surface. However, in some practical and industrial applications, aluminium is free or almost free of oxygen. For instance, in the Hall-Héroult aluminium electrolyse cells, the liquid metal pad is free or nearly of oxygen.

Following the procedure given above, let us formulate and examine the surface tension of aluminium versus T and *x*_*O*_. From the aluminium monoxyde lattice parameters, we estimated, in our prior work, that ${{\rm{\Gamma }}}_{0}^{sat\mathrm{.}}$ = 1.65 × 10^−5^ mol.m^−2 ^^4^. ${x}_{O}^{sat\mathrm{.}}$ as a function of temperature is available in the FactSage thermodynamic software and databases^25^ and it can be represented by the following expression:

13 $${x}_{O}^{sat\mathrm{.}}(T)[ppm]=5.0\times {10}^{-5}+{e}^{11.265-\frac{10964}{T}}$$

According to the most recent assessments^10,26,27^, the surface tension of oxygen saturated liquid aluminium at the melting temperature (933 K) is: ${\sigma }_{O}^{sat\mathrm{.}}$ 0.86 J.m^−2^. Then *σ*(*T*, *x*_*O*_) is predicted.

**Figure 2:**
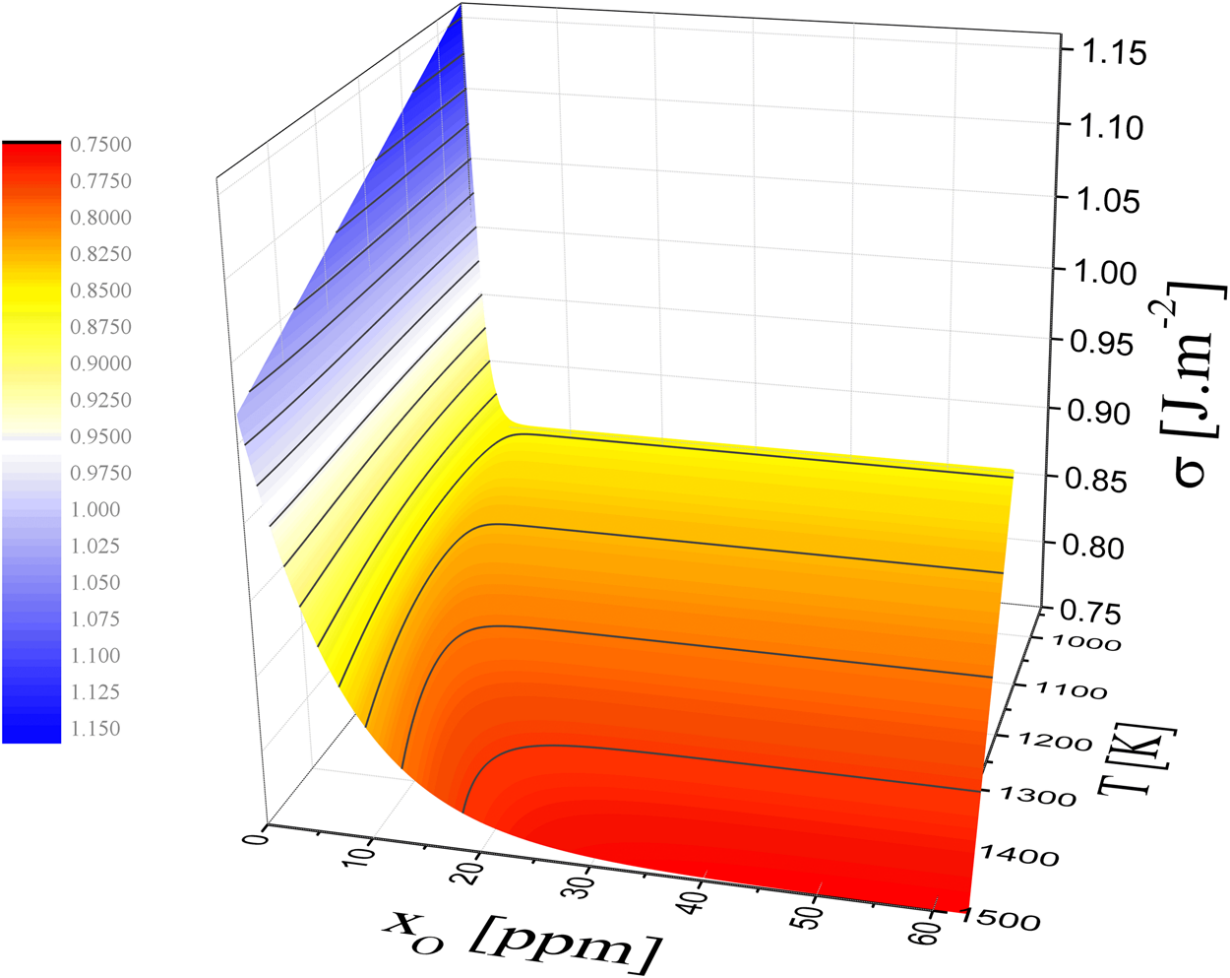
Predicted, via Eq. 8, surface tension of liquid aluminium as a function of temperature and oxygen content. Parameters for Eq. 8: ${{\rm{\Gamma }}}_{O}^{sat\mathrm{.}}$ = 1.65 × 10^−5^ mol.m^−2^, ${\sigma }_{O}^{sat\mathrm{.}}$ = 0.86 J.m^−2^ and ${x}_{O}^{sat\mathrm{.}}$ is a function of temperature calculated by Eq. 13.

First, let us examine the calculated surface tension as a function of both temperature and oxygen content, represented in Fig. 2 at up to 1500 K. The surface tension follows an irregular shape, showing a pronounced asymmetry. At low temperature, close to the melting temperature, the surface tension shows an abrupt decrease with oxygen content and then becomes constant (${\sigma }_{O}^{sat\mathrm{.}}$) whereas at higher temperature, this decrease is smoother and the surface tension reaches ${\sigma }_{O}^{sat\mathrm{.}}$ at higher oxygen level. The linear behaviour of surface tension with temperature is, strictly speaking, true for pure and oxygen saturated liquid metals. The predicted surface tension of pure and oxygen saturated liquid aluminium is given by the following expressions:

14 $$\{\begin{array}{ll}{\sigma }_{pure}(T)=1.16-2.44\times {10}^{-4}\,(T-933) & [J\mathrm{.}{m}^{-2}]\\ {\sigma }_{sat\mathrm{.}}(T)=\mathop{\underbrace{0.86}}\limits_{Exp\mathrm{.}}-1.80\times {10}^{-4}\,(T-933) & [J\mathrm{.}{m}^{-2}]\end{array}$$

**Figure 3:**
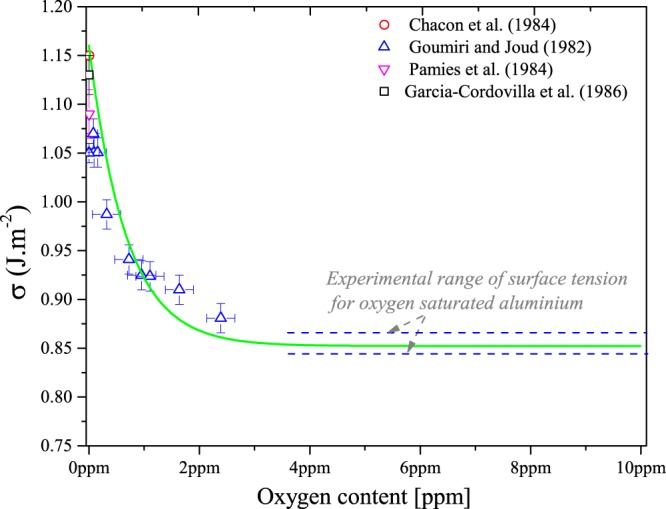
Predicted surface tension of liquid aluminium as a function of oxygen content at melting temperature (933 K) (solid line) in comparison with experimental data at the same temperature (open symbols). References: Chacon *et al*.^28^, Goumiri and Joud^2^, Pamies *et al*.^29^, Garcia-Cordovilla *et al*.^3^. The two dash lines represent the standard deviation of the mean experimental surface tension of oxygen saturated aluminium^1,10,26,27^.

These two equations are parameterized only based on the experimental value of the the surface tension of oxygen saturated metal. In Fig. 3, the surface tension of liquid aluminium at melting temperature (933 K) is represented as a function of oxygen content in comparison with available experimental data. The predicted surface tension of pure Al is in very good agreement with experiments. Our predicted surface tension of 1.16 J.m^−2^ is very close to the experimental value reported by Chacon *et al*.^28^ and Garci-Cardovilla *et al*.^3^ for pure aluminium. It is important to note that in the case of liquid aluminium, the oxygen saturation is less but close to 5 ppm.

**Figure 4:**
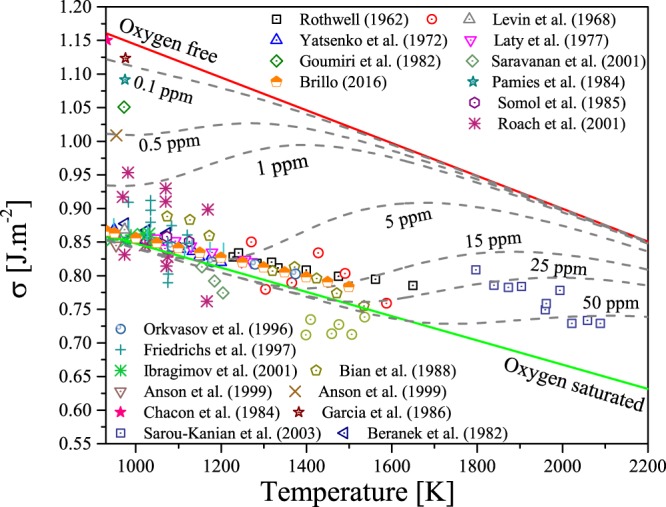
Predicted surface tension of liquid aluminium as a function of temperature for pure (upper solid line), oxygen saturated (lower solid line) and various iso-oxygen contents (dashed lines) from 0.1 to 50 ppm in comparaison with available experimental data (open symbols). Note that data reported by Chacon *et al*.(1984), Pamies (1984) Garcia-Cordovilla *et al*.^3^ Anson *et al*.^31^ are assumed to be for pure or nearly pure liquid aluminium. Experimental data are referenced as follow: Levin *et al*.^44^, Yatsenko *et al*.^45^, Brillo *et al*.^46^, Laty *et al*.^47^, Pamies *et al*.^29^, Somol *et al*.^48^, Sarou-Kanian *et al*.^49^, Rothwell^50^, Goumiri *et al*.^2^, Saravanan *et al*.^30^, Roach *et al*.^32^, Orkvasov *et al*.^51^, Friedrichs *et al*.^52^, Ibragimov *et al*.^53^, Anson *et al*.^31^, Bian *et al*.^54^, Garcia-Cordovilla *et al*.^3^, Beranek *et al*.^55^, Chacon *et al*.^28^.

Let us now discuss the core of this work, the coupling effect between the temperature and the oxygen adsorption at the surface of liquid metals. In Fig. 4 we represent the calculated surface tension of pure liquid aluminium (free of oxygen), oxygen saturated and with various levels of oxygen, from 0.1 ppm to 50 ppm. One can see that the proposed model can predict accurately the temperature dependence of saturated oxygen liquid aluminium. It is interesting to note the data dispersion at melting temperature (933 K). Chacon *et al*.^28^, Pamies *et al*.^29^, Garcia-Cordovilla^3^, Saravanan *et al*.^30^, Anson *et al*.^31^ and to a lesser extent Roach *et al*.^32^ attempted to measure the surface tension of aluminium more or less successfully. Figure 4 helps to understand the discrepancy observed in measurements in terms of oxygen content. For instance, data reported by Garcia-Cordovilla^3^ and Pamies *et al*.^29^ correspond to the surface tension of liquid aluminium containing 0.1 ppm oxygen while those reported by Roach *et al*.^32^ contain about 1 ppm. When considering the reported oxygen level, experimental datasets for supposedly pure liquid aluminium become consistent with each other. The shape of the oxygen content dependence upon the surface tension is particular: it is similar to a cumulative distribution function. In others words, (∂*σ*/∂*x*_*O*_) is described by a peak function of temperature. The peak is positioned where the composition of *x*_*O*_ becomes smaller than ${x}_{O}^{sat\mathrm{.}}$. When the liquid metal is saturated in oxygen, the surface tension obeys $\sigma (T)={\sigma }_{0}^{sat\mathrm{.}}({T}_{m})-{\sigma }_{sat\mathrm{.}}^{^{\prime} }(T)$, but as the temperature rises the oxygen content could, at a certain temperature, be less than the maximum oxygen solubility in the liquid metal. As a result, above this temperature, the liquid metal could start adsorbing oxygen again, leading to an increase of its surface tension.

The good predictive capability of the proposed model when predicting the surface tension of liquid aluminum as a function of both temperature and oxygen content was clear. Naturally, the same theoretical treatment can be employed to predict the surface tension of other transition metals, as functions of T and x_*O*_. The predictive capability of the model is expected to be good for other metals, as the reliability of Eq. 1 has been already proven for a large number of elements in our prior work^4^.

## Conclusion

In conclusion, we presented in this work a thermodynamically self consistent approach to predict the coupling effects between the temperature and adsorbed oxygen upon the surface tension. The model has proven to have a good predictive capability by predicting the temperature dependence of surface tension for several liquid metals (Table 1 and Fig. 1). The proposed method could be useful for current research, for example in Integrated computational materials engineering (ICME) for alloys and process design^33–35^. Indeed, the proposed formalism shows that the surface tension versus T and *x*_*O*_ is intercorrelated with other physical properties: thermal expansion, velocity of sound, heat capacity and oxygen solubility. When building a property database, one can now also consider the surface tension to ensure self consistency between the physical properties. Having more reliable databases lead to better predictions of properties for which few or no experimental data are available. A similar approach was successfully employed to couple thermal thermodynamics and thermal transport properties^36–40^. Lastly, for some liquid metals, due to their significant reactivity with non metallic impurities (O_2_, S, P, C, etc.), it is difficult to measure the surface tension of the pure elements. In a near future, utilising molecular dynamic simulation campaigns based on reliable Modified Embedded Atom Model (MEAM)^41–43^, the surface tension of several liquid metals will be predicted in order to confirm the validity of Eq. 10.

## Data Availability

All data generated or analysed during this study are included in this published article.
